# Laboratory automation, informatics, and artificial intelligence: current and future perspectives in clinical microbiology

**DOI:** 10.3389/fcimb.2023.1188684

**Published:** 2023-06-27

**Authors:** Antonella Mencacci, Giuseppe Vittorio De Socio, Eleonora Pirelli, Paola Bondi, Elio Cenci

**Affiliations:** ^1^ Microbiology and Clinical Microbiology, Department of Medicine and Surgery, University of Perugia, Perugia, Italy; ^2^ Microbiology, Perugia General Hospital, Perugia, Italy; ^3^ Clinic of Infectious Diseases, Perugia General Hospital, Perugia, Italy

**Keywords:** laboratory automation, artificial intelligence, informatics, laboratory workflow, Kiestra, WASPLab

## Abstract

Clinical diagnostic laboratories produce one product—information—and for this to be valuable, the information must be clinically relevant, accurate, and timely. Although diagnostic information can clearly improve patient outcomes and decrease healthcare costs, technological challenges and laboratory workflow practices affect the timeliness and clinical value of diagnostics. This article will examine how prioritizing laboratory practices in a patient-oriented approach can be used to optimize technology advances for improved patient care.

## Introduction

Patterns of infectious diseases have changed dramatically: patients are frequently immunocompromised and often have complicating comorbidities, infections with multi-drug-resistant organisms (MDRO) are a global problem, new antibiotics are available, but it is mandatory to preserve their efficacy. It is estimated that at least 700,000 people die worldwide every year with infections caused by MDRO, and it is predicted that by 2050, 10 million deaths might be due to these organisms (O’Neill, 2016). Administration of rapid, broad-spectrum empiric therapy is essential to improve patient outcome (Levy et al., 2018), but this is often inappropriate (Kumar et al., 2009; Zilberberg et al., 2017). A meta-analysis assessing the impact of antibiotic therapy on Gram-negative sepsis showed that inappropriate therapy was associated with 3.3-fold increased risk of mortality, longer hospitalization, and higher costs (Raman et al., 2015). Thus, rapid, accurate diagnostics are critical for the selection of the most appropriate therapy.

**Figure 1:**
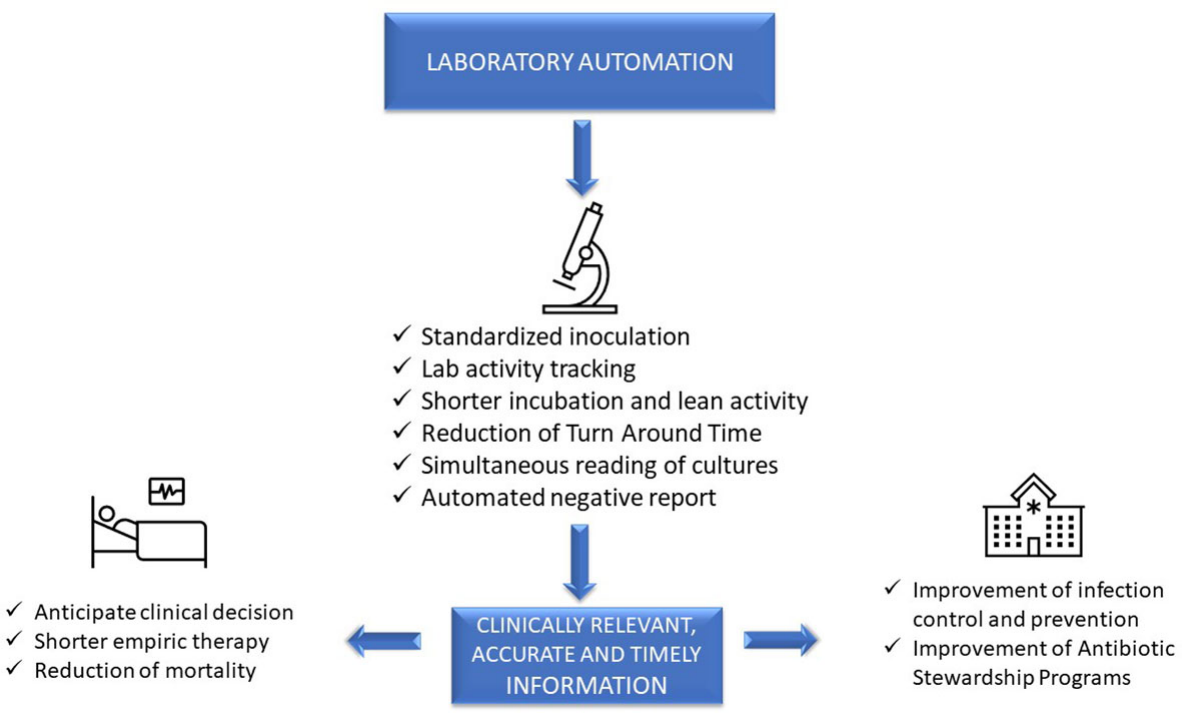
Impact of automation on laboratory, patient, and hospital management.

Advanced, sophisticated technologies such as mass spectrometry and molecular diagnostics are rapidly changing our ability to diagnose infections (Trotter et al., 2019), although they should be viewed as complementary to traditional growth-based diagnostics. Laboratory automation and applications of intelligent use of informatics also have a transformative impact of microbiology diagnostics. These tools have the potential to accelerate clinical decision-making and positively impact the management of infections, improve patient outcome, and facilitate diagnostic and antimicrobial stewardship (AS) programs (Messacar et al., 2017). However, it is a challenge for clinical microbiologists to implement these technologies because it requires changing well-established workflow practices. This paper will focus on the impact of automation and informatics combined with workflow changes on laboratory, patient, and hospital management (**Figure 1**).

## Impact on laboratory management

In clinical microbiology, the term “total laboratory automation” (TLA) is used to describe the automation of the entire diagnostic workflow: inoculation of the agar plates, incubation, reading of culture results, identification (ID), and antimicrobial susceptibility testing (AST). All these steps in a conventional laboratory are performed manually, usually according to a sample-centered approach. At present, two laboratory automation (LA) systems are available: the BD Kiestra™ system (Becton Dickinson, Sparks, MD) and the WASPLab^®^ system (Copan Diagnostics, Murrieta, CA) (Croxatto et al., 2016).

### Inoculation

Quality and precision of inoculation are improved by automation. Instruments work in a standardized and consistent mode, not achievable with a manual procedure, and independent of operator variability. Indeed, LA allows better isolation of colonies compared to manual inoculation, with decreased need of subcultures for follow-up work, mainly AST, resulting in a more rapid report (Croxatto et al., 2015; Burckhardt, 2018). It was found that WASP automated streaking of urines using a sterile loop was superior to manual streaking, yielding a higher number of single colonies and of detected morphologies, species, and pathogens (Quiblier et al., 2016). The BD Kiestra™ system, based on a rolling magnetic bead streaking technology, has been shown to improve the accuracy of quantitative culture results and the recovery of discrete colonies from polymicrobial samples, compared to manual and automated WASP streaking (Croxatto et al., 2015; Iversen et al., 2016). This implies a reduction of bacterial subcultures to perform ID and AST, thus shortening time to results, as evidenced for urines (Croxatto et al., 2017) and both methicillin-resistant *Staphylococcus aureus* (MRSA) and carbapenem-resistant *Enterobacterales* screening samples (Cheng et al., 2020).

### Incubation

Closed incubators with digital imaging of cultures allow more rapid growth than conventional incubators that are opened frequently throughout the day. Moreover, in TLA, plates are fully tracked as long as they stay within the system, so that it is possible to define by hours and minutes incubation times and plate examination in contrast with the traditional system in which incubation times are defined in days. Burckhardt et al. showed that first growth of MRSA, multi-drug-resistant (MDR) Gram-negative bacteria, and vancomycin-resistant enterococci (VRE) on selective chromogenic plates was visible as early as after 4 h of inoculation, although the bacterial mass was not sufficient for follow-up work (Burckhardt et al., 2019). Also, growth of *Escherichia coli*, *Pseudomonas aeruginosa*, *Enterococcus faecalis*, and *S. aureus* on chromogenic plates was 3 to 4 hours faster in the automated than in classic system (Moreno-Camacho et al., 2017). Implementation of BD Kiestra TLA significantly improved turnaround times (TAT) for positive and negative urine cultures (Theparee et al., 2017). Similarly, WASPLab automation enabled a reduction of the culture reading time for different specimens without affecting performances (Cherkaoui et al., 2019). However, minimum incubation times for each type of specimen, for a timely and accurate positive or negative report, are not yet defined and additional studies are needed.

### Reading

Kiestra LA system, through a real-time dashboard, times tasks as they are scheduled. Thus, each technician perfectly knows when the culture plates will be ready for reading and when a follow-up work can be performed. This strongly facilitates laboratory workflow management, avoiding wasted time and allowing results to be delivered to the clinician as soon as possible. In addition, while in the classical system plates are read one-by-one, digital reading allows simultaneous viewing of all the plate images from the same sample, and even of different samples from the same patient. This greatly facilitates and speeds up the interpretation of culture results, either for monomicrobial or polymicrobial infections.

### ID and AST

The implementation of TLA in clinical microbiology has leveraged the advancement brought by matrix-assisted laser desorption/ionization time-of-flight mass spectrometry (MALDI-TOF/MS) (Thomson and McElvania, 2019; Cherkaoui and Schrenzel, 2022). Furthermore, Copan’s TLA has recently integrated an automated device (Colibri™) that can reproducibly prepare the MALDI target for microbial identification. A recent study conducted by Cherkaoui et al. established that the WASPLab coupled to MALDI-TOF/MS significantly reduces the TAT for positive blood cultures (Cherkaoui et al., 2023). Similarly, the BD Kiestra™ IdentifA/SusceptA, a prototype for automatic colony picking, bacterial suspension preparation, MALDI-TOF target plates spotting, and Phoenix™ M50 AST panel preparation, exhibited high ID and AST performances (Jacot et al., 2021). In particular, the IdentifA showed excellent identification rates for Gram-negative bacteria, outperforming manual processing for *Enterobacterales* identification (Jacot et al., 2021), but not for streptococci, coagulase-negative staphylococci (CoNS), and yeasts (Jacot et al., 2021).

Finally, an automated solution for disk diffusion AST was developed and integrated with the Copan WASPLab system. It prepares inoculum suspensions, inoculates culture media plates, dispenses appropriate antibiotic disks according to predefined panels, transports the plates to the incubators, takes digitalized images of the media plates, and measures and interprets the inhibition zones’ diameters. Cherkaoui et al., evaluating 718 bacterial strains including *S. aureus*, CoNS, *E. faecalis*, *Enterococcus faecium*, *P. aeruginosa*, and different species of *Enterobacterales*, found 99.1% overall categorical agreement between this automated AST and Vitek2 (Cherkaoui et al., 2021).

### Artificial Intelligence

The development of intelligent image analysis based on tailored algorithms designed on type of specimens and patient characteristics allows automated detection of microbial growth, release of negative samples, presumptive ID, and quantification of bacterial colonies. This represents a major innovation that has the potential to increase laboratory quality and productivity while reducing TAT (Croxatto et al., 2017). Promising results have been obtained on urine samples, with a 97%–99% sensitivity and 85%–94% specificity by the BD Kiestra system (Burckhardt et al., 2019). By a different approach, the WASPLab Chromogenic Detection Module has developed automated categorization of agar plates as “negative” (i.e., sterile) or “non-negative”, comparing the same plate at time point zero to the plate after the established incubation time. With this system, an optimal diagnostic accuracy in MRSA (Faron et al., 2016a), VRE (Faron et al., 2016b), and carbapenemase-producing Enterobacteriaceae (Foschi et al., 2020) detection has been observed.

### Other functions of LA

LA can greatly facilitate to implement an effective quality system, which is required to ensure that reliable results are reported for patients. LA systems automatically track and record all the useful information for quality control: user credentials, media (lot number and expiration date), inoculation (volumes of samples, patterns, and times of streaking), incubation (atmosphere, temperature, and times) and imaging (digital images of plates and times) data (Dauwalder et al., 2016). Thus, a proper integration of LA with laboratory information system allows for complete traceability of the analytical process, from sample receipt to the final report.

Moreover, the possibility to access and review any taken image represents an invaluable tool from a diagnostic point of view (e.g., comparing morphology of colonies in recent and old samples from the same patient) and also for other activities such as monitoring laboratory quality, teaching, training, and discussing culture results with colleagues and clinicians.

## Impact on patient management

Clinical impact of an assay, a technology, or a modified workflow can be defined based on its added value for patient management. In the case of sepsis, this can be measured as time to targeted therapy and, hopefully, a decrease in the mortality rate. In manual processing laboratories, the activities are performed in batches, usually based on the type of sample and of activity (inoculation, reading, ID, AST, technical validation, and clinical validation), and the results are usually delivered mostly during the morning hours. Indeed, a study evaluating the TAT for positive blood cultures (BC) in 13 US acute care hospitals demonstrated a significant discrepancy between times of BC collection and reporting laboratory test results. While only 25% of specimens were collected between 6:00 a.m. and 11:59 a.m., approximately 80% of laboratory ID and AST results were reported in this time interval (Tabak et al., 2018). This can have a negative impact on septic patient management, delaying clinical decision-making for optimal targeted therapy.

In contrast, in automated laboratories, the activity can be organized according to lean principles, creating a continuous “flow” and producing “just-in-time” results. De Socio et al. evaluated the impact of LA on septic patient management. Positive BC were processed by fully automatic inoculation on solid media and digital reading after 8 h of incubation, followed by ID and AST. The authors found that a reduction of time to report (TTR) of about 1 day led to a significant reduction of the duration of empirical therapy (from approximately 87 h to approximately 55 h) and of 30-day crude mortality rate (from 29.0% to 16.7%) (De Socio et al., 2018).

Therefore, provided that the laboratory is open 24 h a day, or taking advantage of telemedicine systems for clinical validation, LA has a potential great impact on patient management. However, the success of such organization lies in the responsiveness of the medical teams, who should act upon the results soon after delivery by the laboratory (Vandenberg et al., 2020).

## Impact on hospital management

LA can improve the laboratory ability to characterize MDRO and produce quality results, permitting a more standardized workflow, and leaving more time for laboratory staff to focus on second-level phenotypic and/or genotypic tests. Indeed, the large diffusion of MDRO and the expanding spectrum of resistance mechanisms among pathogens pointed out the limitations of commercial routine methods for susceptibility testing of selected antibiotics, increasing the demand for cumbersome and time-consuming reference methods. For example, in the case of MDRO Gram-negative isolates, colistin MIC should be determined by the broth microdilution method (Kulengowski et al., 2019); fosfomycin MIC, by the agar-dilution method (Camarlinghi et al., 2019); and cefiderocol, a novel siderophore-conjugated cephalosporin, by the broth microdilution method using an iron-depleted cation-adjusted Mueller-Hinton broth (Simner and Patel, 2020). Moreover, in the case of detection of uncommon resistance phenotypes, molecular methods, gene sequencing, or other next-generation sequencing methods are often required (Antonelli et al., 2019).

Accuracy is not sufficient *per se* for a result to be useful. Information must be given to clinicians or other healthcare providers (e.g., pharmacists and the patient’s primary care nurse) as quickly as possible. Timely report can affect hospital conditions in at least two ways: permitting to rapidly control the spread of MDRO (contact precautions, investigation of clusters of colonized/infected patients) and reducing the duration of broad-spectrum antibiotic therapy (positive results) or unnecessary empiric antibiotic therapy (negative results).

In a study proposing a cumulative antimicrobial resistance index as a tool to predict antimicrobial resistance (AR) trend in a hospital, a reversion of AR trend was observed in 2018, in comparison with the 2014–2017 period (De Socio et al., 2019). The authors speculate that this could have been a consequence of some changes in the management of infections in their hospital: (i) incubation of all BC within 1 h from collection using satellite incubators, (ii) a significant reduction in TTR after the introduction of molecular technologies and LA, and (iii) an established close collaboration between infectious disease clinicians and clinical microbiologists (De Socio et al., 2019).

Finally, Culbreath et al. demonstrated that the implementation of TLA increased laboratory productivity by up to 90%, while reducing the cost per specimen by up to 47%, provided an excellent elaboration of the efficiencies and cost-savings is achievable by implementation of full LA in the bacteriology laboratory (Culbreath et al., 2021).

## Possible improvements

A detailed wish list of technical issues to be evaluated in order to improve the performance and workflow of LA systems has been recently published (Burckhardt, 2018). Here, we will focus on facts that, in our opinion, could affect laboratory, patient, and hospital management.

To facilitate the reading of the plates according to a patient-centered approach, it would be useful to view the specimens’ Gram stains in the same screen of cultured plates. The images could also be shared with clinicians, improving clinician–microbiologist interplay. Further improvement can be made by automated microscopy systems, which can significantly reduce the workload of the technical staff (Zimmermann, 2021).

The availability of digital images lays the foundation for telebacteriology, intended as the use of digital imaging and file storage for on-screen reading and decision-making (Croxatto et al., 2016). It makes it possible to geographically dissociate plate manipulation from reading and validation of the results. This could promote the microbiologist counseling activity and interaction with clinicians, as the images could be shared between consultants located at different sites. Also, it could support a 24/7 laboratory activity, allowing the plates to be read outside the laboratory in a hub-laboratory or even at home, with follow-up work performed in real time where the plates are incubated.

To make these technological innovations fully operational, a middleware information technology (IT) solution is needed to connect all the laboratory instruments (Zimmermann, 2021).

## Discussion

The main reason to introduce automation in a laboratory is to increase productivity and to face limited budgets and personnel shortages. However, implementation of LA can represent an exceptional opportunity to change laboratory organization, improve quality, and reduce TTR, with a potential positive impact on laboratory, patient, and hospital management.

One of the most relevant innovations of LA regards the reading phase, with the possibility to read simultaneously all the plates inoculated from one of even more samples from the same patient. Moreover, taking advantage of informatics, it is also possible to view patient microbiological, hematological, and even clinical and therapeutic data while reading the plates. This patient-oriented approach provides meaningful clinical interpretation of results and decision-making.

By continuously tracing all the analytical steps, LA ensures that the microbiologist knows in real time the work to be carried out. This concept fully adheres to the so-called “lean” organization that, initially envisaged for industry (Womack et al., 1990), is increasingly applied to healthcare processes. “Lean” means to do only valuable activities, without any delay, avoiding “waste” or unnecessary work. This implies a dramatic revolution in the mentality of microbiologists, transitioning from exclusively sample-centered laboratory work towards a more clinically oriented activity, shortening TTR and prioritizing diagnosis of time-dependent infections. Taking advantage of workflow optimization, about 24 h reduction in TTR has been observed for positive BC processed by LA, with a significant decrease of duration of empirical therapy and mortality (De Socio et al., 2018). Similar results were observed for urines (Yarbrough et al., 2018) and nasal MRSA surveillance (Burckhardt et al., 2018) and all types of specimens (Croxatto et al., 2017; Theparee et al., 2017).

An artificial intelligence algorithm to interpret culture results is another important tool applicable to LA: automated reporting of negative samples can be done without delay and further human assistance, so that clinicians can receive earlier results to rule out MDRO colonization or a urinary tract infection and reduce the need for patient isolation or antibiotic treatment (Faron et al., 2016a; Faron et al., 2016b; Cherkaoui and Schrenzel, 2022).

Beside LA, a variety of technologies are revolutionizing clinical microbiology. These include MALDI-TOF MS (Seng et al., 2009), time-lapse microscopy for ID and phenotypic AST (Charnot-Katsikas et al., 2018), molecular diagnostic tests and syndromic panels (Arena et al., 2017; Trotter et al., 2019), and next-generation sequencing (Mitchell and Simner, 2019; Pitashny et al., 2022). All of them can significantly improve the diagnosis and therapy of infections, but as stated above, they are primarily complementary to culture (Trotter et al., 2019). Thus, in an advanced laboratory, the goal will be to implement the use of all these technologies in a coordinated and timely program of diagnostic stewardship (DS). For example, for active surveillance of MDRO, both molecular and culture methods should be available in the laboratory (Anandan et al., 2015). Indeed, active surveillance of carbapenem-resistant Enterobacteriaceae can limit and prevent their spread and infections, which is crucially relevant to AS (Ambretti et al., 2019). In high-risk patients, rapid molecular methods are more appropriate, but cannot replace culture-based methods, as the latter can detect all types of carbapenem-resistant organisms, perform phenotypic susceptibility testing, and collect and store the isolates (Ambretti et al., 2019). An interesting algorithm, based on a multi-parametric score including clinical, microbiological, and biochemical parameters, has been recently proposed to establish patient priority, including information on infection or colonization by MDRO (Mangioni et al., 2019). It is reasonable to think that by combining DS and AS programs with a strict collaboration between laboratory and clinicians, the impact of modern microbiology on the management of infection can progressively increase.

In this line, rapid and effective communication from laboratory to wards and back is essential for optimal patient care. A recent study showed that many barriers exist, like verbal reporting of results, poorly integrated information systems, mutual lack of insight into each other’s area of expertise, and limited laboratory services (Skodvin et al., 2017). Electronic reporting improves communication between microbiologists and clinical staff, but a sort of alarm for the right physician (i.e., the treating clinician, an infectious diseases specialist, or a sepsis team member) to look up the data immediately should be integrated. Nevertheless, we believe that direct microbiologist/clinician interplay remains crucial for an optimal patient management: positive BC, detection of MDRO, isolation of alert organisms from sterile fluids, and acid-fast bacilli in respiratory samples must be immediately reported to someone who will act on the results.

Moreover, as microbiological methods become increasingly sophisticated, good clinical practice should be for the microbiologist to report the results with comments to facilitate the clinician’s interpretation of the significance of the data (Arena et al., 2017). In our experience, after LA implementation, a closer relationship with clinicians has been established, providing an opportunity to convey insight into microbiology and microbiological work processes to clinical staff. On the other hand, patients are increasingly complex and heterogeneous and management of severe and MDRO infections is challenging, often requiring a multidisciplinary approach for optimal personalized diagnostics and therapy (Tiseo et al., 2022). Therefore, to integrate DS with AS, microbiologists should broaden their knowledge of patient care by working closely with physicians.

Information from the microbiology laboratory is essential for the control and management of infections in a hospital. In particular, timely and accurate data on the antibiotic susceptibility profiles for pathogens isolated from different wards and on MDRO colonization/infection are the basis for setting up hospital infection control and AS programs, which can ultimately affect patient outcome. Unfortunately, laboratories are not always able to provide timely information due to lack of specific expertise, personnel, user-friendly software, and optimized workflow practices. The implementation of LA and informatics can support integration into routine practice monitoring specimens’ quality, isolation of specific pathogens, alert reports for infection control practitioners, and real-time collection of lab trend data, all essential for the prevention and control of infections and epidemiological studies.

In conclusion, timely, accurate, and clinically relevant information is the basis for prevention and treatment of infections. LA and informatics can greatly improve the accuracy of diagnostic procedures, TTR, and laboratory workflow. However, to exploit these technologies for the benefit of the patients, clinical microbiologists need to change their way of working, according to a lean workflow and a patient-centered approach, and their way of thinking, working more closely with clinical staff.

## Data availability statement

The original contributions presented in the study are included in the article/supplementary material. Further inquiries can be directed to the corresponding author.

## Author contributions

Study concept: AM, GD, EC. Critical revision of manuscript: PB, EP. Approval of manuscript: AM, GD, EC, PB, EP. All authors contributed to the article and approved the submitted version.
